# Ghrelin/GHSR Axis Induced M2 Macrophage and Alleviated Intestinal Barrier Dysfunction in a Sepsis Rat Model by Inactivating E2F1/NF-*κ*B Signaling

**DOI:** 10.1155/2023/1629777

**Published:** 2023-12-29

**Authors:** Lei Zhu, Zhimin Dou, Wei Wu, Qiliang Hou, Sen Wang, Ziqian Yuan, Bin Li, Jian Liu

**Affiliations:** Department of Intensive Care Medicine, The First Hospital of Lanzhou University, Lanzhou 730000, China

## Abstract

Sepsis is an inflammatory reaction disorder state that is induced by infection. The activation and regulation of the immune system play an essential role in the development of sepsis. Our previous studies have shown that ghrelin ameliorates intestinal dysfunction in sepsis. Very little is known about the mechanism of ghrelin and its receptor (GHSR) on the intestinal barrier and the immune function of macrophage regulation. Our research is to investigate the regulatory effect and molecular mechanism of the ghrelin/GHSR axis on intestinal dysfunction and macrophage polarization in septic rats. A rat model of sepsis was established by cecal ligation and puncture (CLP) operation. Then, the sepsis rats were treated with a ghrelin receptor agonist (TZP-101) or ghrelin inhibitor (obestatin). The results suggested that TZP-101 further enhanced ghrelin and GHSR expressions in the colon and spleen of septic rats and obestatin showed the opposite results. Ghrelin/GHSR axis ameliorated colonic structural destruction and intestinal epithelial tight junction injury in septic rats. In addition, the ghrelin/GHSR axis promoted M2-type polarization of macrophages, which was characterized by the decreases of IL-1*β*, IL-6, and TNF-*α*, as well as the increase of IL-10. Mechanistically, the ghrelin/GHSR axis promoted E2F2 expression and suppressed the activation of the NF-*κ*B signaling pathway in septic rats. Collectively, targeting ghrelin/GHSR during sepsis may represent a novel therapeutic approach for the treatment of intestinal barrier injury.

## 1. Introduction

Sepsis is a clinical syndrome caused by severe infections that always trigger excessive inflammatory responses [1]. Sepsis, septic shock, and multiple organ dysfunction syndromes (MODS) represent the sequential stages of the disease process [2]. The end stage of MODS is organ failure, which is commonly associated with poor clinical outcomes and a high mortality rate [3, 4]. During sepsis, the body has an uncontrolled inflammatory response to the infection, a large number of inflammatory factors are released, multiple inflammation-related signaling pathways are activated, and finally, MODS are observed [5]. There is an urgent need to find new treatment entry points. Excessive inflammatory reactions affect the intestine during sepsis, and the intestinal mucosal barrier is damaged [6, 7]. A large number of bacteria and endotoxins enter the bloodstream, aggravating the inflammatory response and promoting the process of subsequent organ damage [8, 9]. Therefore, the intestinal tract is not only a damaged organ of sepsis but also an important intermediate link in the development of sepsis into multiple organ dysfunctions, so the protection of the intestinal barrier function in sepsis is very important.

Ghrelin is the endogenous ligand of growth hormone secretagogue, which is derived mainly from X/A cells of the gastric mucosa and is an endocrine peptide containing 28 amino acids [10]. Ghrelin exerts its biological functions mainly by binding to its receptor GHSR [11]. A growing number of studies have shown that ghrelin binds to its receptor and has regulatory effects on growth hormone secretion, feeding, glucose metabolism, cardiovascular system, inflammatory response, cell differentiation, and tumor [12–14]. It has been reported that patients with sepsis had elevated ghrelin levels, which were inversely related to the length of stay in the ICU and SOFA score [15]. Another study found a positive correlation between high serum ghrelin levels and survival in patients with sepsis [16]. In animal models of sepsis, ghrelin could reduce inflammation, improve gastrointestinal blood perfusion, and reduce gastrointestinal injury in sepsis by regulating autophagy [17]. Our previous studies suggested that ghrelin inhibited oxidative stress and intestinal dysfunction to attenuate sepsis by activating SIRT1 and regulating a KLF4/MMP2 regulatory axis [18]. Therefore, it can be speculated that ghrelin may be a new treatment for septic gastrointestinal dysfunction. However, the specific mechanism of its action in sepsis is still unclear.

The transcription factor E2F1, a member of the E2F family, exerts regulatory control over diverse biological processes and plays pivotal roles in numerous diseases, including cancers, obesity, inflammation, and sepsis [19–21]. The bioinformatics study demonstrated that E2F1 was a regulator of differentially expressed genes associated with ferroptosis in sepsis patients [22]. E2F1 was implicated in the regulation of the inflammatory response to toll-like receptor ligands, including lipopolysaccharide (LPS) [23]. The transcriptional response of the coagulation cascade was diminished in E2F1-deficient mice, suggesting that disseminated intravascular coagulation may be a consequence of uncontrolled sepsis [23]. Furthermore, E2F1 could interact with NF-*κ*B forming an E2F1/NF-*κ*B complex. E2F1 inhibited the nuclear translocation of NF-*κ*B p65 by downregulating the phosphorylation level of NF-*κ*B p65, thus deactivating the NF-*κ*B signaling pathway [24]. A previous study revealed a positive correlation between the expression levels of ghrelin and E2F1 during cellular proliferation, suggesting that E2F1 may function as a downstream effector of ghrelin [25]. Meanwhile, in our previous study, we found that ghrelin increased the expression of E2F1, leading to the inhibition of the NF-*κ*B pathway [26]. HDAC5 inhibitor LMK-235 suppressed inflammation in sepsis via the ghrelin/E2F1/NF-*κ*B axis [26].

In the present study, we investigated the effects of ghrelin on colonic inflammation, intestinal barrier function, and immune function in septic rats using ghrelin receptor agonist (TZP-101) and ghrelin inhibitor (obestatin). Furthermore, we explored the regulatory effect of ghrelin on the E2F1/NF-*κ*B signaling pathway in the colon of septic rats.

## 2. Methods and Materials

### 2.1. Animals

Male Sprague–Dawley (SD) rats were purchased from Chengdu Dossy Experimental Animals Co., Ltd. (Chengdu, Sichuan). The feeding environment was 25 ± 1°C, relative humidity was 50%–60%, and light/darkness for 12 h circulation. Rats were allowed to eat and drink freely. Animals and experimental protocol were conducted according to the guidelines and ethical standards of the Animal Care and Use Ethics Committees of Lanzhou University (LDYYLL2021-87).

### 2.2. Sepsis Models

A rat model of sepsis was established by cecal ligation and puncture (CLP) according to a previously described protocol [27, 28]. Rats were fasted for 12 h before the experiment and had free access to water. Anesthesia was induced by intraperitoneal injection of 1% sodium pentobarbital 50 mg/kg. Anesthesia depth was determined by the pinch test. Laparotomy was used to expose the cecum post-general anesthesia. The cecum was gently picked out and ligated with a 4-gauge thread at the 2/3 cecal end to avoid ligation of blood vessels. The cecum was gently perforated once with an 18 G trocar, with two perforations, and little feces was gently extruded to avoid injury to the mesenteric vessels. The cecum was then put back in the abdominal cavity, and the abdominal wall incision was sutured layer by layer to sterilize the incision.

### 2.3. Experimental Design

Thirty-six rats were randomly divided into the normal group, sham operation group, CLP group, ghrelin treatment group, ghrelin + ghrelin receptor agonist (TZP-101) group, and ghrelin + ghrelin inhibitor (obestatin) group, with 6 rats in each group. Rats in the normal group, sham operation group, and CLP group were intraperitoneally injected with 1 ml of normal saline at 2 h after the operation. Rats in the ghrelin group were intraperitoneally injected with ghrelin 20 ng/kg 2 h after the operation, and colon tissues and spleen tissues were collected 12 h after drug intervention. Rats in the ghrelin + TZP-101 group were injected intraperitoneally with ghrelin 20 ng/kg and intravenously with TZP-101 1 mg/kg 2 h postoperation, and colonic tissues and spleen tissues were taken 12 h after drug intervention. Ghrelin + obestatin group rats were injected with ghrelin 20 ng/kg intraperitoneally and obestatin 100 nmol/kg intraperitoneally at 2 h postoperation, and colonic tissues and spleen tissues were taken after 12 h of drug intervention.

### 2.4. Fluorescein Isothiocyanate (FITC) Assay

Rats were fasted for 4 h before the end of the experiment. FITC-dextran (cat. no. 68059; Sigma-Aldrich; Merck KGaA) was orally administered to rats (500 mg/kg body weight, 125 mg/mL). To obtain serum (200 *µ*l), blood samples were centrifuged (2,000 × *g*, 4°C) for 10 minutes after mice were anesthetized with isoflurane. The serum level of FITC-dextran was analyzed by a fluorescence spectrophotometer.

### 2.5. Hematoxylin and Eosin (H&E) Stain

The colonic tissues of rats were collected and fixed in 4% paraformaldehyde overnight, processed, and embedded in paraffin. The tissue sections were stained with hematoxylin and eosin (H&E) to observe the degree of the lesion and inflammatory cell infiltration under a 200x and 400x magnification optical microscope (Olympus BH2, Tokyo, Japan).

### 2.6. Periodic Acid-Schiff (PAS) Stain Reaction Stain

Following the manufacturer's instructions, PAS staining was performed with the PAS Staining Kit (Muto Pure Chemicals, Tokyo, Japan). Colonic tissues were fixed with 10% (w/v) formaldehyde and treated with 1% (w/v) periodic acid for 10 min at room temperature. After the sections were washed three times with distilled water, they were treated with Schiff's reagent for 30 min at 37°C. Then, sections were rinsed using distilled water for 10 min, and nuclei were stained with hematoxylin for 1.5 min. The sections were rinsed using running water and sealed. The sections were examined by light microscopy.

### 2.7. Immunofluorescence (IF) Staining

After the experiments, colonic tissues were dissected and fixed with 4% paraformaldehyde. Paraffin sections of colonic tissues were dewaxed and hydrated. The sections were incubated in QuickBlock™ Blocking Buffer (Beyotime, Shanghai, China) for 30 minutes at room temperature. Then, the sections were incubated with the anti-zonula occludens 1 (ZO-1; Abcam, Cambridge, MA, USA; ab221547; 1/100), anti-claudin-5 (Abcam, Cambridge, MA, USA; ab4648; 1/100), or anti-NF-*κ*B p65 (Cell Signaling Technology, Danvers, MA, USA; 8242; 1/400) at 4°C overnight and washed 3 times with phosphate-buffered saline (PBS). The staining of colonic tissues was observed under a fluorescence microscope BX53 (Olympus, Tokyo, Japan) at 100x or 200 magnification.

### 2.8. Immunohistochemistry (IHC) Stain

Colonic or spleen tissue sections (4 *μ*m) were routinely dewaxed and hydrated with gradient ethanol. The samples were treated with antigen repair solution at 95–99°C for 40 min and cooled at room temperature for 20 min. After washing 3 times, CD86 antibody (ABclonal, Wuhan, China; A2353; 1 : 50) or CD206 (Servicebio, Wuhan, China; GB113497; 1 : 100) was added and incubated overnight at 4°C. Then, the EnVision detection and color development kit was used for DAB color development, hematoxylin restaining, gradient ethanol dehydration, xylene transparency, and treacle sealing for observation. IHC images were evaluated microscopically (BA400Digital, Motic Instruments, Inc., Baltimore, MD, USA).

### 2.9. Enzyme-Linked Immunosorbent Assay (ELISA)

The levels of LPS from rat serum samples and colonic tissues were examined with an ELISA kit (Zhuocai Biological Technology, China) based on the manufacturer's instructions. The tissue was homogenized using a tissue grinder, the homogenate was centrifuged at 5000 × *g* for 10 min, and the supernatant was taken for measurement. The samples were plated for 30 min at room temperature onto a microplate precoated with the antibody specific for LPS. After the immune complex was treated, the absorbance of wells was measured with a microplate reader (SpectraMax Plus 384, USA) at 450 nm wavelength to calculate the sample concentration according to the manufacturer's instructions.

### 2.10. RT-qPCR Assay

The mRNA expression of TNF-*α*, IL-1*β*, IL-6, E2F1, I*κ*B, and p65 was evaluated by using RT-qPCR. For gene analysis, equal amounts of cDNA were added to a reaction mixture containing gene-specific forward and reverse primers deoxynucleotide Taq DNA polymerase and SYBR (Bio-Rad, Hercules, CA) in a reaction mixture. Quantification of cDNA was based on monitoring increased SYBR fluorescence during exponential phase amplification in an RT-qPCR machine (Bio-Rad, Hercules, CA), and the determination of the PCR cycle number at which the amplified product exceeded a defined threshold.

### 2.11. Western Blot Analysis

Colonic or spleen tissue lyses solution was fabricated using RIPA buffer (Signaling Technology, Inc.). The protein concentration was examined by a BCA kit (Sigma-Aldrich; Merck KGaA). Total protein (30 *µ*g/sample) was separated via 10% SDS-PAGE and nitrocellulose membranes. We used 5% nonfat dried milk to block the membranes. The corresponding protein antibodies were as follows: ghrelin (Hua-bio, Chengdu, China; ER63531; 1/1000), GHSR (Abcam, MA, USA; ab85104; 1/1000), IL-1*β* (ABclonal, Wuhan, China; A20529; 1/500), IL-6 (ABclonal, Wuhan, China; A0286; 1/500), IL-10 (ABclonal, Wuhan, China; A2171; 1/500), TNF-*α* (ABclonal, Wuhan, China; A20851; 1/500), and *β*-actin (Boster, Wuhan, China; BM0627; 1/1000). Then, the membrane washing was performed with Tris-buffered saline/0. 1% Tween (TBST) and incubated for 1.5 hours with an HRP Goat anti-Rabbit IgG (Abcam, ab6721). The band visualization was carried out using the ECL system (Affinity Biosciences, Cincinnati, Ohio, USA), and as an internal control, *β*-actin was used.

### 2.12. Statistical Analysis

The results of the experiments were statistically analyzed using SPSS analytical statistical software. The experimental results were expressed using the mean ± standard deviation (SD). One-way analysis of variance (ANOVA) was used for statistical analysis. A difference of *p*  <  0.05 was defined as significant.

## 3. Results

### 3.1. Ghrelin/GHSR Axis Ameliorated Colonic Structural Destruction in Septic Rats

**Figure 1:**
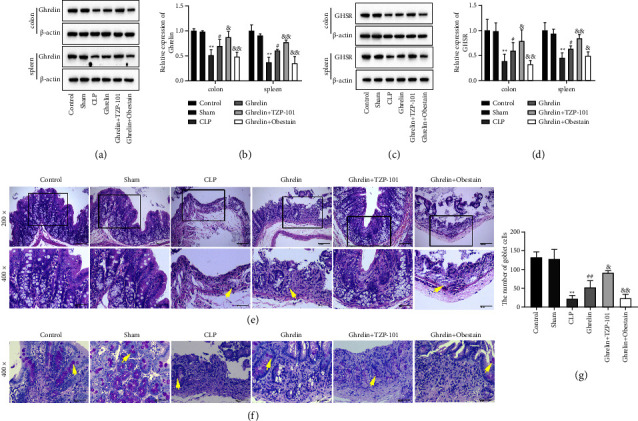
Ghrelin/GHSR axis ameliorated colonic structural destruction in septic rats. (a–d) The expression of ghrelin and GHSR in the colon and spleen tissues of septic rats was determined by Western blot. (e) Colon histopathological changes were analyzed by H&E stain (200x and 400x magnification). (f) Representative photomicrographs of PAS-stained colon tissues (400x magnification). (g) The goblet cell count of the colonic tissue. ^*∗∗*^*P* < 0.01 (vs sham group), ^#^*P* < 0.05 (vs CLP group), ^##^*P* < 0.01 (vs CLP group), ^&^*P* < 0.05 (vs ghrelin group), and ^&&^*P* < 0.01 (vs ghrelin group).

The growth hormone secretagogue receptor (GHSR) was characterized as a member of the G-protein-coupled receptor family and as a newly founded receptor of ghrelin [29]. Our results found that the expression of ghrelin and GHSR was decreased in the colon and spleen of septic rats which was induced by ghrelin treatment (Figures 1(a)–1(d)). Meanwhile, TZP-101 further enhanced ghrelin and GHSR expressions in the colon and spleen of septic rats and obestatin showed the opposite results (Figures 1(a)–1(d)). These results suggest that the ghrelin/GHSR axis may regulate the gastrointestinal function and immune function in septic rats.

A histological examination was performed after staining with H&E or PAS. CLP treatment caused extensive exfoliation of crypt epithelial cells, crypt destruction, and loss of glandular structure (Figure 1(e)). The ghrelin treatment group ameliorated the disruption of colonic structure caused by CLP treatment (Figure 1(e)). In addition, compared with the ghrelin-treated group, ghrelin and TZP-101 cotreatment further reduced the disruption of colonic structure. However, obestatin hindered the ameliorative effect of ghrelin on the colon of septic rats (Figure 1(e)). As shown in Figures 1(b) and 1(c), the colons of CLP mice had fewer goblet cells compared with the control and sham groups. Ghrelin treatment enhanced the number of goblet cells compared with the CLP group (Figures 1(f) and 1(g)). Furthermore, ghrelin and TZP-101 cotreatment further increased the number of goblet cells compared with the ghrelin-treated group. The cotreatment of ghrelin and obestatin showed the opposite results (Figures 1(f) and 1(g)). The higher number of goblet cells in the ghrelin and ghrelin + TZP-101 group may have protected sepsis rats from CLP-induced injury.

### 3.2. Ghrelin/GHSR Axis Attenuated Intestinal Epithelial Tight Junction Injury in Septic Rats

**Figure 2:**
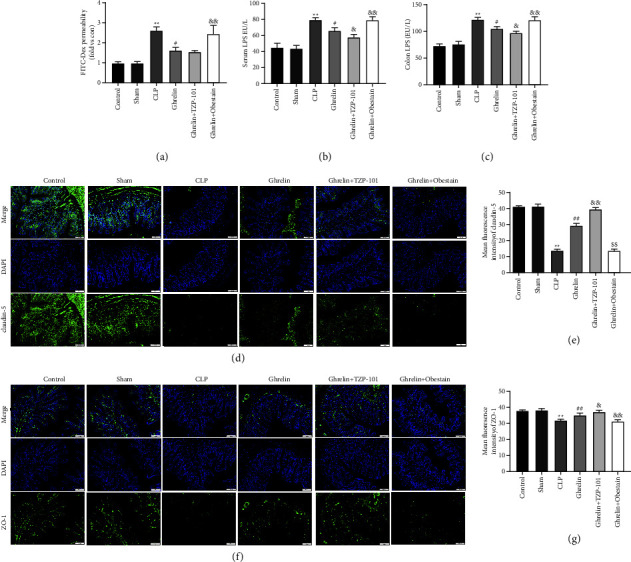
Ghrelin/GHSR axis attenuated intestinal epithelial tight junction injury in septic rats. (a) FITC-dextran serum levels were measured 4 hours after gastric administration to determine intestinal permeability. Higher serum FITC-dextran levels indicated more intestinal permeability. Serum LPS (b) and colon LPS (c) were assayed by ELISA. (d–g) Immunofluorescence (IF) analysis determining the expression and distribution of claudin-5 and ZO-1 in colon tissues of septic rats (100x magnification). ^*∗∗*^*P* < 0.01 (vs sham group), ^#^*P* < 0.05 (vs CLP group), ^&^*P* < 0.05 (vs ghrelin group), and ^&&^*P* < 0.01 (vs ghrelin group).

The intestinal permeability of septic rats was increased, and the content of FTC-glucan in serum was significantly higher than that in control and sham groups (Figure 2(a)). Ghrelin treatment decreased the content of FTC-glucan in the serum of septic rats, which was reversed by obestatin (Figure 2(a)). LPS, a component of the intestine that triggers inflammation, plays a role in mucosal barrier function [30, 31]. Then, the levels of serum LPS (Figure 2(b)) and colonic LPS (Figure 2(c)) were determined. As shown in Figures 2(b) and 2(c), in the CLP model, the LPS contents in the serum and colon were significantly increased as compared to the control and sham groups. On the contrary, ghrelin treatment effectively decreased the levels of colonic LPS and serum LPS in comparison to the CLP group (Figures 2(b) and 2(c)). Furthermore, compared with the ghrelin-treated group, ghrelin and TZP-101 cotreatment further reduced the levels of LPS in the colon and serum (Figures 2(b) and 2(c)). Cotreatment with ghrelin and obestatin significantly hindered the lowering effect of ghrelin on LPS levels (Figures 2(b) and 2(c)).

IF stain was used to detect the expression of intracellular scaffolding protein claudin-5 (Figures 2(d) and 2(e)) and ZO-1 (Figures 2(f) and 2(g)) in the colons of septic rats. The fluorescence intensity of claudin-5 was diminished in CLP-treated mice, similar to changes reported in ZO-1 (Figures 2(d)–2(g)). The redistribution and depletion of these two proteins were restored by ghrelin (Figures 2(d)–2(g)). Meanwhile, ghrelin and TZP-101 cotreatment further enhanced the expression of claudin-5 and ZO-1 compared with the ghrelin group (Figures 2(d)–2(g)). The cotreatment of ghrelin and obestatin showed the opposite results (Figures 2(d)–2(g)). Together, these findings indicated that the protective effect of ghrelin on intestinal epithelium may be derived from its role in improving compromised tight junctions.

### 3.3. Ghrelin/GHSR Axis Inhibited Colonic Inflammation in Septic Rats

**Figure 3:**
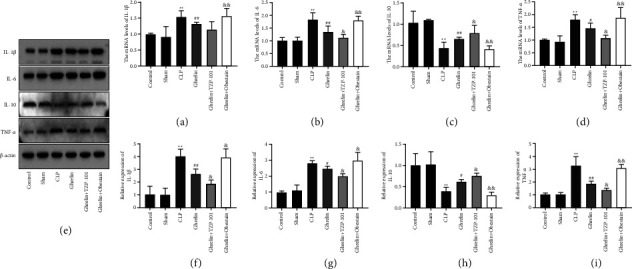
Ghrelin/GHSR axis inhibited colonic inflammation in septic rats. (a–d) The mRNA levels of IL-1*β*, IL-6, IL-10, and TNF-*α* in colon tissues of septic rats were tested by RT-qPCR. (e–i) Protein levels of IL-1*β*, IL-6, TNF-*α*, and IL-10 were assayed by Western blot analysis. ^*∗∗*^*P* < 0.01 (vs sham group), ^#^*P* < 0.05 (vs CLP group), ^##^*P* < 0.01 (vs CLP group), ^&^*P* < 0.05 (vs ghrelin group), and ^&&^*P* < 0.01 (vs ghrelin group).

We examined if inflammatory cytokines are known to mediate colonic inflammation, such as IL-1*β*, IL-6, TNF-*α*, and IL-10. As shown in Figures 3(a)–3(d), CLP treatment increased the mRNA expression of L-1*β*, IL-6, and TNF-*α*, as well as decreased the mRNA expression of IL-10. Ghrelin treatment reduced the mRNA expression of L-1*β*, IL-6, and TNF-*α*, as well as enhanced the mRNA expression of IL-10 (Figures 3(a)–3(d)). Furthermore, ghrelin and TZP-101 cotreatment further reduced the levels of IL-1*β*, IL-6, and TNF-*α*, as well as enhanced the levels of IL-10 compared with the ghrelin-treated group (Figures 3(a)–3(d)). The cotreatment of ghrelin and obestatin showed the opposite results (Figures 3(a)–3(d)). Protein levels of IL-1*β*, IL-6, TNF-*α*, and IL-10 were assayed by Western blot analysis. As shown in Figures 3(e)–3(i), compared with the sham group, CLP induction resulted in elevations of IL-1*β*, IL-6, and TNF-*α* as well as a reduction in IL-10 expression (Figures 3(e)–3(i)). Ghrelin treatment decreased the protein levels of IL-1*β*, IL-6, and TNF-*α* and increased the protein levels of IL-10 (Figures 3(e)–3(i)). Compared to the ghrelin-treated group, the ghrelin- and TZP-101-treated group exhibited decreased IL-1*β*, TNF-*α*, and IL-6 levels and increased levels of IL-10 (Figures 3(e)–3(i)). The cotreatment of ghrelin and obestatin showed the opposite results (Figures 3(e)–3(i)).

### 3.4. Ghrelin/GHSR Axis Promoted M2-Type Polarization of Macrophages

**Figure 4:**
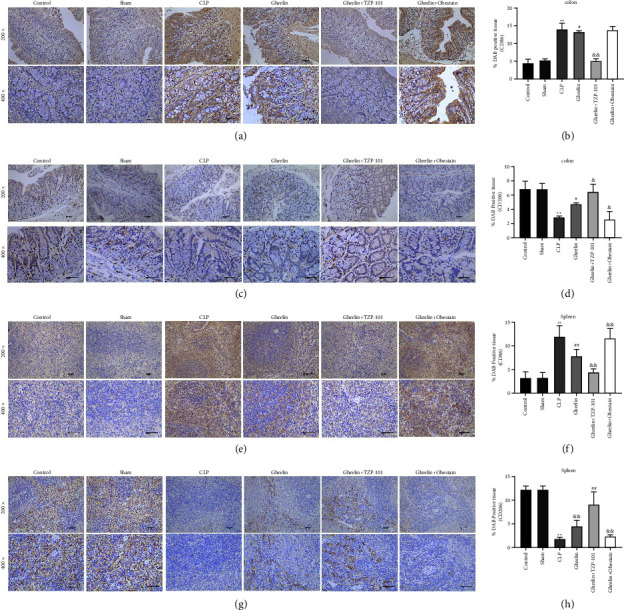
Ghrelin/GHSR axis promoted M2-type polarization of macrophages. (a–d) The colon samples of septic rats were subjected to an immunohistochemical (IHC) stain with a primary antibody against CD86 and CD206 (200x and 400x magnification). (e–h) The spleen tissues were stained with anti-CD86 and anti-CD206 and analyzed by IHC stain (200x and 400x magnification). ^*∗∗*^*P* < 0.01 (vs sham group), ^#^*P* < 0.05 (vs CLP group), ^##^*P* < 0.01 (vs CLP group), ^&^*P* < 0.05 (vs ghrelin group), and ^&&^*P* < 0.01 (vs ghrelin group).

We then examined the effect of the ghrelin/GHSR axis on macrophage polarization in the colon and spleen of septic rats. CD86 and CD206 are specific markers of M1 macrophages and M2 macrophages, respectively. IHC stain showed that the expression of CD86 was significantly increased and that of CD206 was significantly decreased in the colon and spleen of septic rats (Figures 4(a)–4(h)). In addition, CD86 expression was decreased and CD206 expression was increased in the colon and spleen of ghrelin-treated septic rats (Figures 4(a)–4(h)). TZP-101 reduced and obestatin enhanced CD86 expression in the colon and spleen of septic rats compared with the ghrelin alone treatment group (Figures 4(a)–4(h)). As expected, TZP-101 promoted and obestatin inhibited CD206 expression in the colon and spleen of septic rats compared with the ghrelin alone treatment group (Figures 4(a)–4(h)). These results suggest that the ghrelin/GHSR axis promoted M2-type polarization of macrophages in the colon and spleen of septic rats.

The ghrelin/GHSR axis activated E2F1 expression and suppressed the activation of the NF-*κ*B signaling pathway in septic rats.

**Figure 5:**
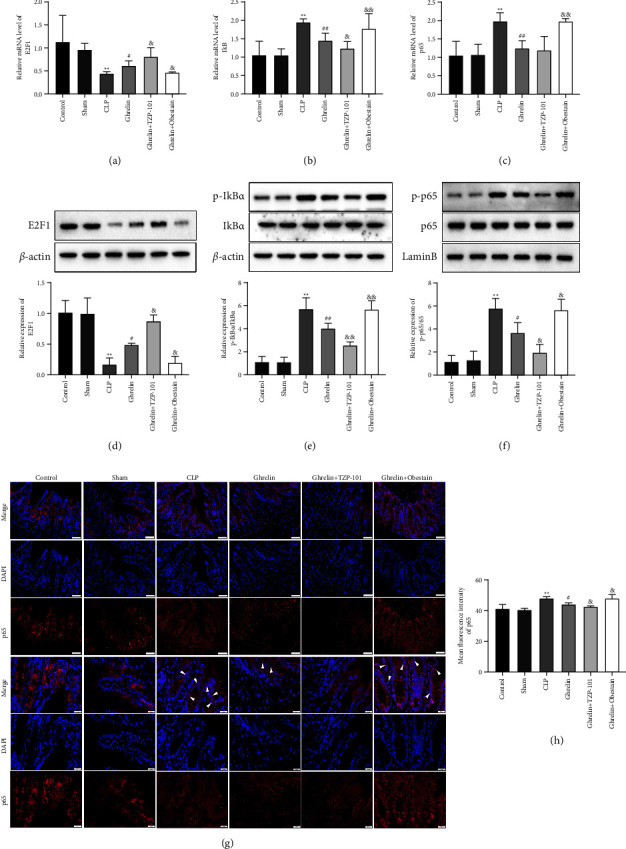
Ghrelin/GHSR axis activated E2F1 expression and suppressed the activation of the NF-*κ*B signaling pathway in septic rats. (a–c) The mRNA levels of E2F1, I*κ*B, and p65 in colon tissues of septic rats were detected by RT-qPCR. (d–f) Proteins in cytoplasmic or nuclear extracts were determined using Western blot. ((g), (h)) Nuclear translocation of p65 was determined using immunofluorescence. The scale labels shown are 50 *µ*m and 20 *µ*m. The white arrows indicate the nuclear translocation of p65. ^*∗∗*^*P* < 0.01 (vs sham group), ^#^*P* < 0.05 (vs CLP group), ^##^*P* < 0.01 (vs CLP group), ^&^*P* < 0.05 (vs ghrelin group), and ^&&^*P* < 0.01 (vs ghrelin group).

Finally, we explored the molecular mechanisms by which ghrelin ameliorates gastrointestinal dysfunction in sepsis. The mRNA level of E2F1 was decreased and the mRNA levels of I*κ*B and NF-*κ*B p65 were increased in the colon of septic rats (Figures 5(a)–5(c)). Furthermore, ghrelin promoted the E2F1 mRNA level and inhibited the mRNA levels of I*κ*B and p65 compared with the CLP group (Figures 5(a)–5(c)). TZP-101 treatment increased E2F1 mRNA level and inhibited I*κ*B mRNA level compared with the ghrelin alone treatment group. Obestatin treatment showed the opposite results (Figures 5(a)–5(c)). Detection of protein levels was performed with Western blot analysis. As shown in Figures 5(d)–5(f), the results showed that the expression of E2F1 was downregulated and the phosphorylation of I*κ*B and p65 was upregulated in the colon of septic rats. Ghrelin activated E2F1 expression and suppressed the phosphorylation of I*κ*B and p65 (Figures 5(d)–5(f)). In addition, TZP-101 treatment increased E2F1 expression and inhibited the expression of I*κ*B in the cytoplasm and p65 in the nucleus compared with the ghrelin alone treatment group (Figures 5(d)–5(f)). On the contrary, obestatin resulted in decreased E2F1 expression, leading to increased phosphorylation of I*κ*B and p65 compared with the ghrelin alone treatment group (Figures 5(d)–5(f)). Finally, IF staining was performed to examine the nuclear localization of p65. Compared with the CLP group, nuclear localization of p65 was downregulated significantly by ghrelin, and TZP-101 inhibited and obestatin promoted this effect (Figures 5(g)–5(h)).

## 4. Discussion

Severe sepsis of abdominal origin leads to the impairment of intestinal barrier integrity, which is mainly manifested as enhanced intestinal mucosal permeability, intestinal mucosal perfusion disorder, tissue edema, and bacterial translocation [32, 33]. The destruction of intestinal epithelial cell function in patients with severe abdominal sepsis is a key factor causing septic shock and multiple organ dysfunction syndrome [34]. The structure of the intestinal epithelium with its tight junction disposal allows only the passage of very tiny molecules, preventing bacterial or macromolecular (e.g., LPS) transport [35]. Under normal circumstances, the intestinal tract absorbs nutrients while maintaining bacteria within the intestinal lumen. In sepsis, the changes in the intestinal permeability to macromolecular follow the same time course as bacterial overgrowth and increased toxin production, and the intestine may allow increased bacterial infiltration into mesenteric lymph nodes (MLNs) and other extraintestinal sites (e.g., spleen, lung, liver, and blood) [7, 36, 37]. Our results showed that crypt epithelial cells were shed and crypt architecture was disrupted in the intestinal tissues of septic rats, which were reversed by treatment with ghrelin and ghrelin receptor agonist TZP-101. Furthermore, paracellular bacterial transport may also be facilitated by changes in intestinal epithelial cell structure, particularly involving tight junctions [38]. The tight junction is mainly composed of various tight junction proteins, such as occludin, claudin-5, and ZO-1 [39, 40]. The study suggested that serum ZO-1 could serve as a robust biomarker for assessing gut barrier dysfunction in sepsis [41]. In a study investigating the impact of berberine pretreatment on sepsis, occludin, ZO-1, and claudin-4 were found to exhibit decreased expression levels following CLP in rats [42]. Meanwhile, irisin upregulated occludin and ZO-1 in lung tissues of sepsis-induced acute lung injury rats [43]. To the best of our knowledge, this study represented a pioneering demonstration that ghrelin and TZP-101 could enhance claudin-5 and ZO-1 levels in the intestinal tissue of septic rats. Thus, our results suggested that the ghrelin/GHSR axis attenuated intestinal barrier permeability and intestinal epithelial tight junction injury in septic rats, which was consistent with our previous findings [18].

The dysregulation of inflammation serves as the fundamental mechanism underlying sepsis pathogenesis and persists throughout sepsis [44]. The anti-inflammatory properties of ghrelin have been demonstrated in several *in vivo* studies. In septic mice, ghrelin decreased cerebral edema and improved the blood-brain barrier integrity by decreasing inflammation [45]. Another study reported that ghrelin improved ventricular peak systolic pressure and cardiac contractility of CLP rats by reducing proinflammatory cytokines [46, 47]. In CLP-induced septic mice, ghrelin administration improved inflammatory cytokine levels, kidney function, and arterial blood pressure [48]. On the other hand, TNF-*α* and IL-6 levels in rats were decreased following CLP surgery by administration of ghrelin [49]. After 12–24 h after CLP, ghrelin significantly improved survival in mice and reduced clinical parameters and histopathological scores of sepsis, thus demonstrating its effects on late mediators of inflammation [50]. Similarly, we found that in CLP rats, ghrelin administration produced significant inhibition of the release of TNF-*α*, IL-6, and IL-1*β*, as well as increased levels of IL-10 in colon tissues. These findings align with our previous study which found that ghrelin ameliorated CLP-induced sepsis in mice by reducing inflammation and weight loss [51]. In conclusion, our study further clarified the inhibitory effect of ghrelin on intestinal inflammation in septic rats.

The main contributors to inflammatory cytokine production in sepsis are reported to be macrophages [52]. Macrophages possess the ability to perform chemotaxis, antigen presentation, phagocytosis, and pathogenic bacteria eradication, while also serving the crucial role of regulating the inflammatory response to uphold homeostasis, thereby establishing their significance as a pivotal defense mechanism within the human body [53, 54]. Macrophages are believed to be extremely plastic [55]. In the early stages of sepsis, which is characterized by a systemic inflammatory response, infection triggers the differentiation of macrophages to M1-type macrophages, inducing the secretion of a large number of inflammatory factors that participate in the pathogenic bacterial killing and clearance process [56]. In the later stage of sepsis characterized by immunosuppression, macrophages differentiate into M2-type macrophages, which highly express anti-inflammatory cytokines and participate in tissue repair to control excessive inflammatory response [57]. Thus, targeted regulation of macrophage polarization may offer new treatment modalities for sepsis. Zhang et al. [58] recently found that monocyte chemotactic protein (MCP)-induced protein 1 (MCPIP1) regulated M2-type macrophage polarization through inhibition of the JNK/c-Myc signaling pathway, thus reducing sepsis-induced acute lung injury and mortality. Chronic *Schistosoma japonica* (SJ) infection had a protective effect in septic mice by enhancing M2-type macrophage polarization and inhibiting M1-like macrophage polarization [59]. In addition, T-cell immunoglobulin mucin 3 (Tim-3) inhibited M1-type macrophage polarization, thereby reducing proinflammatory activity in the early stage of LPS-induced sepsis [60]. However, in the later stage of sepsis, a decrease in Tim-3 expression inhibited M2-like macrophage polarization and promoted M1-type macrophage polarization, respectively [60]. In our study, the ghrelin/GHSR axis inhibited M2-type polarization of macrophages and promoted M2-type polarization of macrophages in the colon and spleen of septic rats. Ultimately, these reduced anti-inflammatory factors release and avert inflammatory insults.

Furthermore, our study creatively identified the potential of E2F1/NF-*κ*B signaling to inhibit sepsis-induced intestinal dysfunction *in vivo*. The correlation between p65 and E2F1 has been previously confirmed in human fibroblasts, where this physical interaction hinders the expression of E2F-responsive genes [61]. Similarly, E2F1 has been documented to hinder the antiapoptotic NF-*κ*B signaling pathway by diminishing the levels of TNF receptor-associated factor 2, a key activator of NF-*κ*B. This inhibition occurs either through competitive binding with p50 for RelA/ p65 in murine fibroblasts or by suppressing I*κ*B phosphorylation [62–64]. Likewise, the interplay between E2F1 and NF-*κ*B was reported to regulate inflammation in human cardiac cells [65]. E2F1 inhibited the binding of NF-*κ*B p65 to the ICAM-1 promoter and eliminated the antitumor immune effect of ICAM-1 against prostate cancer cells [66]. It was reported that NF-*κ*B is a pleiotropic transcription factor implicated in the regulation of sepsis and septic shock [67]. Inhibition of miR-155 alleviated inflammation and intestinal barrier dysfunction by suppressing NF-*κ*B signaling in mice with sepsis [68]. miR-199a-5p exacerbated intestinal barrier dysfunction by promoting the activation of the NF-*κ*B pathway [69]. In addition, the study found that silencing of miR-31 protected against intestinal barrier dysfunction by inhibiting the NF-*κ*B/HIF-1*α* pathway and targeting HMOX1 during sepsis [70]. Furthermore, somatostatin (SST) repaired sepsis-induced intestinal barrier dysfunction through suppression of NF-*κ*B signaling [71]. Notably, our data suggested that the ghrelin/GHSR axis promoted E2F1 expression and suppressed the NF-*κ*B signaling pathway in septic rats. To the best of our knowledge, the present study is the first to report the inhibitory effect of the ghrelin/GHSR axis on the E2F1/NF-*κ*B signaling pathway in a rat model of sepsis, which implies that the protective effect of ghrelin/GHSR axis on sepsis-induced intestinal barrier dysfunction was mediated by eliminating the activity of E2F1/NF-*κ*B signaling pathway.

Taken together, our results reveal a modulatory effect of the ghrelin/GHSR axis on intestinal barrier function and immune function in sepsis. The ghrelin/GHSR axis increased intestinal barrier function by eliminating tight junction damage. Furthermore, the ghrelin/GHSR axis suppressed the CLP-induced inflammatory response by promoting M2-type polarization of macrophages. The mechanisms underlying the effects of the ghrelin/GHSR axis may be related to the regulation of the E2F1/NF-*κ*B signaling pathway. Thus, the ghrelin/GHSR axis can be a potential therapeutic target for treating human sepsis.

## Data Availability

The datasets used or analyzed during the current study are available from the corresponding author on reasonable request.
